# Marginal adaptation and bond strength of novel resin-based sealers compared with traditional epoxy-resin sealers

**DOI:** 10.6026/973206300220245

**Published:** 2026-01-31

**Authors:** Waleed Ali F Alshadidi, Adamya Shakti Nigam, Shankar S, Nanditha S.K, Anamika Sinha, Pushpendu Singh Yadav, Miral Mehta

**Affiliations:** 1 Department of Endodontics, Mazayalian Dental complex Clinic Abha, Saudi Arabia; 2 Department of Conservative Dentistry and Endodontics, LNCT Dental College and Research Centre, Bhopal, Madhya Pradesh, India; 3 Department of Conservative Dentistry and Endodontics, BGS Global Institute of Dental Sciences, Bangalore, Karnataka, India; 4 Department of Conservative Dentistry and Endodontics, NSVK Sri Venkateshwara Dental College and Hospital, Bangalore, Karnataka, India; 5 Department of Conservative Dentistry and Endodontics, Bharati Vidyapeeth (Deemed to be University) Dental College and Hospital, Navi Mumbai, India; 6 Department of Periodontics, Rungta college of dental sciences and research, Kohka-kurud Road, Bhilai, Chhattisgarh, India; 7 Department of Pediatric and Preventive Dentistry, Karnavati School of Dentistry, Karnavati University, Gandhinagar, Gujarat, India

**Keywords:** Endodontic sealers, resin-based sealers, epoxy-resin, bond strength, push-out test, marginal adaptation, scanning electron microscopy

## Abstract

The long term quality of the treatment of root canals is reached when a hermetic seal is obtained, but the traditional epoxy-resin sealers tend to demonstrate poor adhesion and poor adaptation. Therefore, it is of interest to compared resin-based sealer made of methacrylate to the existing epoxy-resin and calcium silicate-based sealer in terms of bond strength and marginal adaptation. Ninety human teeth were extracted, obturated, and tested in terms of push-out bond strength and marginal gaps that were assessed by SEM. The experimental sealer of methacrylate resin showed high bond strength and marginal adaptation compared with all groups (p=0.001). Thus, we show that it has a better sealing capacity and may lead to better clinical outcome in endodontic treatment.

## Background:

The success of endodontic therapy could neither be achieved without comprehensive chemomechanical debridement of the root canal space and three-dimensional obturation which prevent reinfection and offer long hermetic sealing [1]. Although gutta-percha may be maintained as the gold standard core material, endodontic sealers play the important role of sealing the irregularities, accessory canals and gaps between the core material and the root canal walls [2]. Sealer-dentin interface is one potential route of microleakage of bacteria; thus, ideal marginal adaptation and powerful adhesion are crucial qualities of endodontic sealers. The most commonly studied and commonly used epoxy-resin based sealers, specifically AH Plus (Dentsply Sirona), have been used in decades of clinical use [3]. AH Plus exhibits desirable characteristics such as dimensional stability, low solubility, sufficient working time and acceptable biocompatibility [4]. Nevertheless, epoxy-resin sealers are clinically successful and have low adhesion ability to root dentin, and their adhesion is based mostly on mechanical interlocking but not on means of bonding [5]. This mechanical retention can be undermined by sclerosis of dentinal tubules, artifacts of smear layer, and water pollution. The recent dental materials developments have resulted in the introduction of new type resin-based sealers that include methacrylate monomers, bioactive substances, and adhesive technologies [6]. These materials intend to attain high levels of bonding to radicular dentin using chemical methods of adhesion just like those used in coronal adhesive dentistry. Methacrylate resin sealers theoretically have superior penetration of dentinal tubules resulting in formation of resin tags that are advantageous in increasing micromechanical retention [7]. There are also formulations, which use self-etching primers or functional monomers that also chemically react with hydroxyapatite and have the potential to form a hybrid layer at the sealer-dentin interface.

The availability of bioceramic sealers with calcium silicates has also become an alternative category of resin-based material, exhibiting bioactivity, alkaline pH and dimensional stability, by hygroscopic expansion [8]. The materials have demonstrated good potential of biocompatibility and sealing capacity although their adhesive nature is under investigation [9]. The most common test used to determine the endodontic sealer bond strength to root canal dentin has been the push-out test, which provides benefits over the traditional shear and tensile tests because it more accurately models the distribution of stresses in clinical practice [10]. This ordeal is an evaluation of the force needed to eject the filling substance out of a slice in a root canal, which can give quantitative data regarding interfacial adhesion. The addition of marginal adaptation with the help of scanning electron microscopy (SEM) enables to visually check and quantify interfacial gaps and offer qualitative and quantitative data on sealer adaptation [11]. Although there is an increasing trend in the use of resin based endodontic sealers, there is little and inconsistent comparative data on the marginal adaptation and bond strength of the sealers in comparison to already known epoxy-resin based products [12]. Therefore, it is of interest to evaluate marginal adaptation and bond strength of novel resin-based sealers compared with traditional epoxy-resin sealers.

## Materials and Methods:

## Study design and ethical approval:

This comparative *in vitro* experimental study was conducted at the Department of Conservative Dentistry and Endodontics between March 2024 and December 2024.

## Sample size calculation:

Sample size was calculated using G*Power software version 3.1.9.7 based on preliminary data from a pilot study. Assuming an effect size of 0.40, alpha error of 0.05, and power of 80% for detecting significant differences in bond strength among three groups using one-way ANOVA, a minimum sample size of 26 specimens per group was determined. To account for potential specimen loss during preparation, 30 teeth per group were selected, yielding a total sample of 90 teeth.

## Tooth selection and preparation:

Ninety freshly extracted intact single-rooted human permanent teeth (maxillary and mandibular incisors, canines, and premolars) extracted for periodontal or orthodontic reasons were collected. Teeth were stored in 0.1% thymol solution at 4°C and used within three months of extraction.

## Inclusion criteria: 

(1) Single root canal confirmed radiographically; (2) fully formed apices; (3) absence of calcifications, resorption, or cracks; (4) root curvature less than 10 degrees; (5) root canal diameter compatible with size 15 K-file insertion to working length.

## Exclusion criteria:

(1) Previous endodontic treatment; (2) presence of caries, cracks, or fractures; (3) internal or external resorption; (4) severe root curvature; (5) immature apex; (6) calcified canals.

Selected teeth were decoronated perpendicular to the long axis using a diamond disc under water cooling to obtain standardized root segments of 16 mm length. Working length was established 1 mm short of the apical foramen using size 15 K-file.

## Root canal preparation:

Root canals were prepared using rotary nickel-titanium instruments (ProTaper Next, Dentsply Sirona) following manufacturer's recommendations to final apical preparation of size X4 (40/.06 taper). Irrigation was performed with 3 mL of 2.5% sodium hypochlorite between each instrument change. Final irrigation protocol consisted of 5 mL 17% EDTA for 3 minutes, followed by 5 mL 2.5% sodium hypochlorite, and final rinse with 5 mL sterile saline. Canals were dried using sterile paper points corresponding to the master apical file size.

## Experimental groups and root canal obturation:

Specimens were randomly allocated using computer-generated randomization into three groups (n=30):

Group A (Control): AH Plus sealer (Dentsply DeTrey, Konstanz, Germany) - two-component epoxy-resin based sealer mixed according to manufacturer's instructions.

Group B: EndoSequence BC Sealer (Brasseler USA, Savannah, GA) - premixed calcium silicate-based bioceramic resin sealer.

Group C (Experimental): Methacrylate resin-based experimental sealer containing Bis-GMA, TEGDMA, UDMA, photoinitiators, and glass filler particles (65% by weight), with incorporated 10-MDP adhesive monomer for chemical bonding to hydroxyapatite.

Single-cone technique of obturating root canals with gutta-percha cones (size 40/.06, Diadent, Seoul, Korea) and corresponding sealers was used. Lentulo spiral were used at low speed to introduce the sealsers into the canal and the master cone was coated with sealer and inserted into the working length. Heated instrument was used to remove excess gutta-percha and the access cavities were filled temporarily (Cavit-G, 3M ESPE). The specimens kept their moisture to a hundred percent at 37°C during the 14 days to enable full setting of sealers.

## Push-out bond strength testing:

After the storage, the roots were sawed perpendicular to the long axis using a precision cutting machine (Isomet 1000, Buehler, USA) in water cooling to achieve 2- mm-thick slices. Three cross sections of each root were made of coronal (2-4 mm coronal end), middle (7-9 mm), and apical (13-15 mm) third. Slice thickness was checked with the help of digital caliper. All the slices were marked and kept in distilled water awaiting testing. The push-out bond strength test was carried out in a universal testing machine (Instron 3345, Instron Corporation, USA) that had load cell of 500 N. The different slices were placed on a specifically designed metallic platform fitted with a center hole that had free flow of the filling substance. Compressive load was applied to the apico-coronal direction using stainless steel cylindrical plungers of the correct diameters (1.0mm, 0.8mm and 0.6mm coronal middle and apical slices respectively) to the crosshead at a speed of 0.5mm/min until dislodgement occurred. The failure load was measured in the Newtons.

The push out bond strength (MPa) was determined as follows:

Bond strength (MPa) = F / A

Where F is the maximum failure load (N) and A is bonding area (mm 2) and is computed as:

A = π (R + r) x h

x R is the coronal radius, x r is the apical radius, and x h is the slice thickness (2 mm). The radii were measured with digital caliper under the magnification of x 4.

## Failure mode analysis:

After the push-out testing, fractured surfaces of each specimen were observed under stereomicroscope (Leica M80, Germany) at x 40 magnification to observe the mode of failure to be as follows: (1) Adhesive failure at sealer-dentin interface, (2) Cohesive failure within sealer, (3) Cohesive failure within dentin, (4) Mixed failure (adhesive and cohesive).

## SEM marginal adaptation assessment:

Another ten specimens of each group were made in the same protocol of scanning electron microscopy analysis. Diamond disc was used to longitudinally section roots in order to expose obturated canal. The specimens were dried by the use of increasing ethanol concentration (70 percent, 80 percent, 90 percent, 95 percent as well as 100 percent) at high concentration, gold-sputtered and scanned under a scanning electron microscope (JEOL JSM-6510LV, Japan) at a magnification of 1000 times. Three standardized images were taken at three levels, coronal, middle and apical thirds of every specimen. Image J software was used to measure the marginal gap width (distance between sealer and canal wall) at 10 predetermined points per image (National Institutes of Health, USA). Gap width means of each specimen were averaged.

## Statistical analysis:

The analysis of data was carried out with the help of SPSS Statistics version 25.0 (IBM Corporation, USA). Shapiro-Wilk test and homogeneity of variance was done using Levene test to test normal distribution. Two-way ANOVA were used to analyze the data of push-out bond strengths to compare the influence of the type of sealer and area of the root canal, and then the pair-wise comparison was conducted using the post-hoc test of Tukey. In comparison of marginal gap measurements, one-way ANOVA was used with post-hoc test by Tukey. Chi- square test was used to analyse failure mode distribution. The set statistical significance was p<0.05.

## Results:

All 270 root slices (90 specimens x 3 slices) were successfully prepared and tested. Data demonstrated normal distribution and homogeneity of variance, justifying parametric testing. Two-way ANOVA revealed statistically significant effects of sealer type (p<0.001), root canal region (p<0.001), and their interaction (p=0.018) on push-out bond strength. Overall mean push-out bond strength values are presented in Table 1. Group C (experimental methacrylate resin-based sealer) demonstrated significantly higher bond strength (8.47 ± 1.82 MPa) compared to both Group A (5.93 ± 1.45 MPa) and Group B (6.21 ± 1.67 MPa) (p<0.001). No significant difference was observed between Groups A and B (p=0.394). Values expressed as mean ± standard deviation. Different superscript capital letters (A, B) in the same column indicate statistically significant differences among sealers (p<0.05). Different superscript lowercase letters (a, b, c) in the same row indicate statistically significant differences among root canal regions (p<0.05). Bond strength values decreased progressively from coronal to apical regions in all groups (p<0.001). The coronal third demonstrated significantly higher bond strength (8.28 ± 2.11 MPa) compared to middle (6.85 ± 1.76 MPa) and apical thirds (5.48 ± 1.48 MPa). Post-hoc pairwise comparisons confirmed statistically significant differences between all regional pairs (p<0.001). Failure mode distribution differed significantly among groups (χ^2^=28.45, p<0.001) as presented in Table 2. Adhesive failures at the sealer-dentin interface predominated in Groups A (73.3%) and B (66.7%), indicating weak interfacial bonding. Mixed failures (combination of adhesive and cohesive) were most common in Group C (56.7%), suggesting stronger interfacial adhesion. Cohesive failures within sealer material occurred infrequently across all groups (6.7-10.0%). No cohesive failures within dentin were observed. Regional analysis revealed that adhesive failures were most prevalent in apical regions (68.9%) compared to middle (55.6%) and coronal regions (48.9%) (χ^2^=11.23, p=0.024), correlating with the reduced bond strength observed apically. SEM examination of 30 specimens (10 per group) yielded 90 images for quantitative gap analysis. Representative SEM images demonstrated distinct interfacial characteristics among groups. One-way ANOVA revealed statistically significant differences in marginal gap width among sealers (F=89.34, p<0.001). Group C demonstrated significantly superior marginal adaptation with mean gap width of 2.34 ± 0.89 µm compared to Group A (5.67 ± 1.94 µm) and Group B (4.82 ± 1.56 µm) (p<0.001). No significant difference existed between Groups A and B (p=0.128). The percentage of well-adapted areas (defined as gap width <3 µm) was markedly higher in Group C (78.6%) compared to Groups A (34.2%) and B (42.8%). Gap width increased progressively from coronal to apical regions in all groups, consistent with bond strength findings. The apical third demonstrated significantly larger gaps compared to coronal regions (p<0.001). SEM images of Group C revealed intimate adaptation with minimal interfacial gaps and evidence of sealer penetration into dentinal tubules, forming resin tag-like structures. Groups A and B showed variable adaptation with frequent gap formation and separation at the interface Table 3.

## Discussion:

The paper presents a comparative *in vitro* experimental evaluation of endodontic sealer push-out bond strength and marginal adaptation of an experimental endodontic sealer made with methacrylate resin, relative to established epoxy-resin and calcium silicate-based bio-ceramic sealers. The major results indicated that the experimental resin-based sealer had considerably stronger bond strength and marginal adaptation than both traditional materials, and bond strength gradually declined between coronal and apical levels irrespective of the sealer. The obtained results rejected the null hypothesis of the first and third one and accepted the null hypothesis of the second one, which discussed the regional differences. It is possible to suggest that the much stronger push-out bond of the experimental resin-based sealer made of methacrylate (8.47 MPa) than of AH Plus (5.93 MPa) could be explained by underlying changes in the adhesion mechanisms. Maharti *et al.* reported that AH Plus, an epoxy resin-based sealer, adheres through mechanical interlocking within dentinal tubules rather than chemical bonding with dentin [13]. Conversely, the polymeric materials that comprise methacrylate with functional monomers like 10-MDP can form chemical bonds with the hydroxyapatite crystals in dentin resulting to a more stable and durable interface [14]. Moreover, resin monomers have lower viscosity levels, which promote deeper penetration of tubular resin, creating large resin tags, which increase micromechanical retention. All these mechanisms lead to the high adhesive performance in our study. The values of the push-out bond strengths of AH Plus in the current experiment (5.93 MPa average) are correlated with the values documented financeable in the literature (between 4.5 and 7.2 MPa) [3] in comparable experimental setups. This consistency makes our methodology valid and aids the reliability of our findings. Bio ceramic sealer (Endosequence BC sealer) showed similar effective performance to AH Plus (6.21 Mpa) which is consistent with the recent comparative study [8]. In spite of some benefits of bioceramic materials being bioactive and releasing calcium ions, their bonding mechanism is not the same as that of resin-based materials. The bonding of bio ceramics is mainly by interfacial mineralization and chemical bonding with dentin which can take long setting times and optimum moisture levels.

The systematic reduction in the strength of the bonds between coronal and apical regions which is found in all groups supports the aforementioned results and is attributable to various anatomical and technical factors [15, 16]. The apical third poses more problems such as a loss in the number and diameter of dentinal tubules, formation of more sclerosis, inaccessibility to irrigate and remove debris, and poor ability to control moisture optimally. All these factors undermine sealer penetration, interfacial contact, and eventual bond strength. Also increased internal stresses occur in the apical region during obturation as a result of taper incompatibility and constrained geometry with the potential to cause microdefects at the interface. Failure mode analysis helped in having a better insight into the nature of interfacial bonding. Group A and B (73.3% and 66.7%, respectively) have the highest number of adhesive failures meaning that the least strong point was at the sealer- dentin interface and not the material cohesive strength itself. On the other hand, the mixed failures were more in Group C (56.7 percent) and this indicates that interfacial adhesion was close to or more superior to the cohesive strength of the sealer, which is a better bonding condition. This trend has been linked to high levels of clinical performance and microleaks resistance [17]. The SEM results about the marginal adaptation proved to support the push out test results showing much smaller interfacial gaps with the experiment based resin-based sealer (2.34 µm) in comparison to the traditional materials. The diameter of the gaps that go above 3-5 µm is clinically significant because it can allow bacteria to enter the tissue and destroy the apical seal [18]. In 78.6 of the tested areas (compared to control groups), the experimental sealer produced well-adapted interfaces (gap <3 µm), significantly higher. SEM images showed the formation of resin tags in the dentinal tubules, which is the evidence of the effective penetration and micromechanical interlocking. This high adaptability of resin materials can be explained by the fact that they have a reduced film thickness and wettability characteristics as well as volumetric stability on polymerization in case they are well formulated [19]. It is necessary to note that resin-based sealers have proven to be better adhesive agents; however, its success in clinics is determined by several factors such as proper selection of cases, close attention to moisture, and their compatibility with obturation methods. The nature of resin materials is also technique-sensitive and moisture-intolerant, which may inhibit their application in problematic clinical situations, characterized by constant exudate or bleeding [20]. Also, the dimensional stability and hydrolytic degradation resistance of long-term in the humid root canal setting have not been thoroughly studied and need further research with prolonged aging regimes and clinical trials. This study has the limitations of being *in vitro*, which is not biologically as realistic as the root canal system because it does not take into account such factors as tissue fluid dynamics, pulpal pressure, and bacterial challenge. Determining the use of relatively young dentin teeth which have been extracted recently might not reflect old dentition with hardened tubules which are often seen in clinical practice. The obturation method used, the single-cone method, though standardized as research protocol, might affect the sealer volume and distribution in a different manner than warm vertical compaction or other methods can. Moreover 14 days storage is enough to set the initial sealer, but it does not discuss the long-term aging, as well as resistance to degradation. Artificial aging conditions recreating years of clinical service, sealing ability by evaluating microleakage or bacterial penetration techniques, biocompatibility and tissue reaction studies, and finally, randomized controlled clinical trials should all be well-designed in future studies to confirm *in vitro* results [13, 15].

## Conclusion:

The experimental methacrylate resin-based sealer showed better adhesion and marginal adaptation of the conventional epoxy-resin and bioceramic sealers meaning that it has a better sealing potential. Its superior adhesive characteristics imply clinical potentials of long-term root canal obturation. Long-term and clinical research is still needed to verify these results and enable the normal use of clinical practice.

## Figures and Tables

**Table 1:** Mean push-out bond strength (MPa) according to sealer type and root canal region

**Sealer Group**	**Coronal Third**	**Middle Third**	**Apical Third**	**Overall Mean**
Group A (AH Plus)	7.12 ± 1.38^Aa^	5.85 ± 1.24^A^^b^	4.82 ± 1.21^A^^c^	5.93 ± 1.45^A^
Group B (BC Sealer)	7.45 ± 1.52^Aa^	6.18 ± 1.45^A^^b^	5.00 ± 1.38^A^^c^	6.21 ± 1.67^A^
Group C (Experimental)	10.28 ± 1.94^Ba^	8.52 ± 1.62^B^^b^	6.61 ± 1.45^B^^c^	8.47 ± 1.82^B^
Regional Mean	8.28 ± 2.11^a^	6.85 ± 1.76^b^	5.48 ± 1.48^c^	6.87 ± 2.01

**Table 2:** Distribution of failure modes among experimental groups

**Failure Mode**	**Group A (AH Plus) n=90**	**Group B (BC Sealer) n=90**	**Group C (Experimental) n=90**	**Total n=270**
Adhesive (sealer-dentin)	66 (73.3%)	60 (66.7%)	30 (33.3%)	156 (57.8%)
Cohesive (within sealer)	6 (6.7%)	9 (10.0%)	9 (10.0%)	24 (8.9%)
Cohesive (within dentin)	0 (0%)	0 (0%)	0 (0%)	0 (0%)
Mixed	18 (20.0%)	21 (23.3%)	51 (56.7%)	90 (33.3%)
χ^2^=28.45, p<0.001. Values expressed
as frequency (percentage)

**Table 3:** Marginal adaptation assessment: mean gap width (µm) at sealer-dentin interface

**Parameter**	**Group A (AH Plus)**	**Group B (BC Sealer)**	**Group C (Experimental)**	**p-value**
Coronal third	4.23 ± 1.67^a^	3.58 ± 1.34^a^	1.45 ± 0.72^b^	<0.001*
Middle third	5.89 ± 1.88^a^	4.91 ± 1.52^a^	2.18 ± 0.84^b^	<0.001*
Apical third	6.89 ± 2.15^a^	6.01 ± 1.79^a^	3.39 ± 1.12^b^	<0.001*
Overall mean	5.67 ± 1.94^A^	4.82 ± 1.56^A^	2.34 ± 0.89^B^	<0.001*
Percentage of well-adapted areas (gap <3 µm)	34.20%	42.80%	78.60%	<0.001*
Maximum gap observed (µm)	11.2 ± 2.8	9.8 ± 2.3	5.9 ± 1.6	<0.001*
Values expressed as mean ± standard deviation.
Different superscript letters in the same row
indicate statistically significant differences
(Tukey's post-hoc test, p<0.05).
*Statistically significant (p<0.05).

## References

[1] Olivieri JG (2024). J Dent..

[2] Somani R (2019). Int J Clin Pediatr Dent..

[3] Lyu M (2023). Int J Oral Sci..

[4] Xiao Z (2025). Materials Tody Nano..

[5] Kim HR (2017). Restor Dent Endod..

[6] Donnermeyer D (2019). Odontology..

[7] Blaszczyk-Pospiech A (2025). Materials (Basel)..

[8] Al-Haddad A, Che Ab Aziz ZA. (2016). Int J Biomater..

[9] Di Girolamo S (2022). J Maxillofac Oral Surg..

[10] Ozer H (2023). Front Dent Med..

[11] Polineni S (2016). J Conserv Dent..

[12] Collado-González M (2017). J Endod..

[13] Maharti ID (2025). J Conserv Dent Endod..

[14] Etiennot L (2025). J Adhes Dent..

[15] Almousa AM (2025). J Pharm Bioallied Sci..

[16] Mishra P (2017). J Clin Exp Dent..

[17] Porto ARNP (2025). Restor Dent Endod..

[18] Arikatla SK (2018). J Conserv Dent..

[19] Alvarez-Vasquez JL (2024). Dent Med Probl..

[20] Kangseng T (2025). Int Endod J..

